# Retinal small vessel dilatation in the systemic inflammatory response to surgery

**DOI:** 10.1038/s41598-022-17467-7

**Published:** 2022-08-02

**Authors:** Alexander Grogan, Karen Barclay, Deb Colville, Lauren Hodgson, Judy Savige

**Affiliations:** 1 Department of Medicine (Melbourne Health and Northern Health), The University of Melbourne, Parkville, VIC 3050 Australia; 2 Department of Surgery (Northern Health), University of Melbourne, Epping, VIC 3076 Australia; 3 Centre for Eye Research Australia, Royal Victorian Eye and Ear Hospital, East Melbourne, VIC 3101 Australia

**Keywords:** Biomarkers, Cardiology, Risk factors

## Abstract

Retinal microvascular calibre has been proposed as a predictor of cardiac events. Surgery is a major stimulus for inflammation which potentially affects small vessel calibre. This study examined the effects of surgery on retinal, and thus systemic, small vessel size, and the potentially confounding effect of surgery when retinal vessel calibre is used to predict cardiac risk in hospital patients. Consecutive participants were recruited from a preoperative assessment clinic at a teaching hospital. They provided demographic and clinical details, and underwent retinal imaging before and again, within 3 days after surgery, with a non-mydriatic retinal camera. Images were graded for vessel calibre using semi-automated software based on the Parr-Hubbard formula with Knudtson’s modification (IVAN, U Wisconsin). Differences were examined using Fisher’s exact test or a paired t-test, and calibre determinants identified from univariate and multiple linear regression analysis (STATA version 11.2). Sixty-eight participants (23 men, 34%) with a mean age of 55 ± 14.5 years, were recruited. Fourteen (21%) underwent a laparotomy which was considered major surgery and 54 (79%) had Other surgery. Mean C-reactive protein (CRP) levels increased post-operatively from 7.8 ± 20.2 mg/L to 43.9 ± 55.1 mg/L (p < 0.01), and mean serum albumin decreased from 38.9 ± 4.4 g/L to 33.9 ± 5.5 g/L (p < 0.01). Mean central retinal arteriole and venular equivalent calibre (CRAE, CRVE) increased post-operatively (142.4 ± 13.3 µm to 146.4 ± 13.0 µm, p < 0.01 and 213.1 ± 16.8 µm to 217.9 ± 18.3 µm, p < 0.01, respectively). The systemic microvasculature dilates post-operatively possibly secondary to inflammation and endothelial dysfunction. These changes were present within 3 days of surgery and may confound the use of small vessel calibre to predict cardiac risk in surgical inpatients. Microvascular dilatation in response to other inflammatory stimuli such as pneumonia is a known potential confounder in hospital patients.

## Introduction

Inflammation induced by infection, trauma, or systemic disease results in the characteristic features of redness, heat, swelling, pain and loss of function to remove the source of irritation, allow repair and restore homeostasis^1^.

Surgery is a powerful stimulus for inflammation, with its increased production of inflammatory mediators, and consequent changes in vascular caliber, flow and permeability^2–5^. Acute inflammation normally resolves spontaneously through self-regulation^6^ but if inflammatory mediator levels remain elevated over time, small vessels may be damaged through the systemic inflammatory response syndrome (SIRS)^7,8^.

Changes in the systemic small vessels can be demonstrated in the retinal microvasculature and are associated with cardiovascular events, diabetic dysfunction, cerebral lacunar infarction and progressive renal failure^9–12^. Retinal imaging is a non-invasive, reproducible and inexpensive method that demonstrates small vessel structure and pathology^13^. However retinal calibre also reflects multiple systemic factors^14^. Thus small vessel narrowing occurs with male sex, age, hypertension, and renal impairment^9,10,13–16^. Dilatation occurs with inflammatory diseases such as diabetes, dyslipidaemia, obesity, and active inflammatory diseases such as rheumatoid arthritis as well as in chronic obstructive pulmonary disease^17–19^. Calibre is also dynamic, and increases with better hypertension control, and in response to the volume loss after an episode of haemorrhage^20^. Arteriolar and venular calibre are typically interrelated^21^, and when one increases or decreases the other does too, but arteriolar calibre usually changes less.

Small vessel changes have also been used as predictors of cardiac and cerebrovascular disease^9,12,17^. Microvascular retinopathy is associated with cardiac events^22^. Diabetic microvascular retinopathy is associated with cardiac risk, renal failure and peripheral neuropathy^10,23–25^. Retinal arteriolar narrowing is a risk factor for cardiac events in men^26,27^, and venular dilatation for cardiac events in women^28^.

Inflammation has been associated with retinal venular dilatation in other inflammatory diseases, but the effect of surgery on the systemic microvascular calibre has not been investigated previously^18^. The inflammation associated with surgery potentially contributes to inflammation-induced vascular disease but also represents a confounder when microvascular calibre is used to assess cardiac risk. This study examined small vessel caliber before and after surgery, and the determinants of this change.

## Participants and methods

### Study design

This was an observational cohort study of consecutive surgical patients recruited from the preoperative assessment clinic at a metropolitan teaching hospital over a three month period.

Study participants were assisted to complete a questionnaire that included demographic and clinical details, and then underwent retinal imaging, and review within three days of surgery at which time they had repeat retinal imaging. Surgery was considered major when it involved a laparotomy, and Other when there was no laparotomy. Routine post-operative care included monitoring of fluid status, blood pressure and in the case of diabetics, blood sugar levels. Repeat retinal imaging was undertaken as soon as individuals were able to mobilise after surgery. Deidentified retinal images were examined for arteriole and venular calibre at a grading centre by a trained grader. Ocular axial length was not measured because its effect on vessel calibre is uncertain^29^.

The hypothesis for this study was that the inflammation of surgery resulted in systemic microvascular calibre dilatation including in the retinal small vessels. The primary aim of this study was to demonstrate the effect of surgery on the retinal small vessel calibre and the determinants of any change.

Inclusion criteria were participants aged at least 18 years who were booked to undergo surgery. Exclusion criteria were ungradable retinal images. All individuals attending the pre-operative assessment clinic were first approached to participate and then underwent repeat retinal photography post-operatively prior to discharge. The protocol was not altered and no preliminary analysis was performed prior to study completion.

The study was approved by the Human Research Ethics Committee at Northern Health, according to the Principles of the Declaration of Helsinki, and all participants provided written, informed consent to participation.

### Data collection

Participants were assisted to complete a structured questionnaire that included demographic and clinical details including vascular risk factors (hypertension, diabetes, dyslipidaemia, smoking history). The diagnoses of hypertension and diabetes were based on previous clinician diagnoses. BP measurements and relevant laboratory test results were obtained from participant electronic medical records, both at the pre-assessment clinic and on the day of repeat retinal imaging.

### Retinal imaging and grading

Participants underwent digital retinal photography using a non-mydriatic retinal camera (CR5-45NM, CANON, Japan). At least two images were taken of each eye, one centred on the fovea and the other on the optic disc.

Retinal vessel calibre was measured by a trained grader on coded images using a detailed standardized protocol and computer-assisted semi-automated imaging software (IVAN, version 1.30, University of Wisconsin, Madison, WI)^30,31^. The software measured arteriolar and venular calibre of the 6 largest vessels to produce the central retinal artery and vein equivalents (CRAE and CRVE) using Knudtson’s revision of the Hubbard Parr formula^31^.

### Statistical analysis

Data with dichotomous outcomes were summarized in contingency tables as frequencies and percentages. Continuous data were described as mean ± standard deviation. Fisher’s exact test was used to compare percentages and student’s t-test for continuous variables (SPSS, Chicago). Multiple stepwise linear regression analysis was performed to examine the determinants of retinal vessel calibre. Results were considered significant where p < 0.05 and the CI did not include 1.00.

## Results

### Participant characteristics

Seventy-three subjects were recruited, and five were excluded because of ungradable retinal images from cataract (n = 2) or because the imaging software could not identify the largest vessels for measurement (n = 3).

The cohort thus comprised 68 participants, with 45 (66%) women and 23 (34%) men with an overall mean age of 55.1 ± 14.5 years (range 24–83) (Table 1). Twenty-six (38%) had hypertension, with 23 (32%) treated with one or more antihypertensive medications. Sixteen (24%) had dyslipidaemia, 6 (9%) were diabetic and 33 (49%) were current or former smokers (range 0.5–90 pack years).

**Table 1:** Participant characteristics.

Characteristic	Total patients (n = 68)
Age (mean ± SD, years)	55.1 ± 14.5
Males	23 (34%)
**Hypertension**	26 (38%)
Number of antihypertensive medications	1.7 ± 1.2
Dyslipidaemia	16 (24%)
eGFR < 90 (mL/min/1.73m^2^)	25 (37%)
**Diabetes**	6 (9%)
Duration (mean ± SD, years)	8.7 ± 2.2
HbA1C levels (mean ± SD, mmol/L)	6.5 ± 0.7
Random blood sugar (mean ± SD, mmol/L)	6.4 ± 1.6
**Smokers (former and current)**	33 (49%)
Average pack years (mean ± SD)	23.5 ± 39.8
Cancer	17 (25%)
**Type of surgery**	
**Major surgery (Laparotomy)**	14 (21%)
Bowel resection	9 (14%)
Hartmann's reversal	2 (4%)
Abdominal cyst excision	1 (1%)
Rectal fistula repair	1 (1%)
**Other types of surgery**	54 (79%)
Laparoscopic cholecystectomy	16 (24%)
Hernia repair	17 (25%)
Total/hemithyroidectomy	18 (25%)
Haemorrhoidectomy	1 (1%)
Rectopexy/rectal tumour excision	1 (1%)
Breast cancer resection	2 (4%)

Fourteen (21%) underwent major surgery requiring a laparotomy, and 54 (79%) had Other surgery (Table 1). Seventeen operations (25%) were for the treatment of cancer.

None of the participants undergoing surgery had any post-operative complications by day 3. None had a period of hypotension beyond the observation period in recovery, none required admission to the intensive care unit and none had an infection or pulmonary embolus diagnosed by the time of the second retinal photograph.

### Pre- and post-operative changes

#### Clinical features

The average mean arterial pressure decreased post-operatively, from 90 to 84 mmHg (p < 0.01) (Table 2). Mean CRP levels increased from 7.8 to 43.9 mg/L (p < 0.01). Serum albumin decreased from a mean of 38.9 to 33.9 g/L (p < 0.01), and haemoglobin concentrations fell post-operatively from a mean of 138.7 to 125.5 g/L (p < 0.01). Renal function (eGFR) did not change (p = 0.78).

**Table 2:** Pre- and post-operative biomarker and vessel calibre changes.

Characteristic	Pre-operative (n = 68)	Post-operative (n = 68)	p-value	Difference, 95% CI
Mean arterial pressure (mean ± SD, mmHg)	90 ± 9	84 ± 10	**< 0.01**	− 6, − 3 to − 9
CRP (mean ± SD, mg/L)	7.8 ± 20.2 (n = 49)	43.9 ± 55.1	**< 0.01**	36.1, 19.9 to 52.8
Serum albumin (mean ± SD, g/L)	38.9 ± 4.4 (n = 64)	33.9 ± 5.5 (n = 48)	**< 0.01**	− 5.0, − 3.4 to − 6.1
Haemoglobin level (mean ± SD, g/L)	138.7 ± 16.8 (n = 67)	125.5 ± 15.5 (n = 51)	** < 0.01**	− 13.2, − 15.7 to − 9.1
eGFR (mean ± SD mL/min/1.73m^2^)	84.8 ± 1 0.2	85.3 ± 9.0 (n = 49)	0.78	0.5, − 3.1 to 4.1
Retinal arteriolar calibre (CRAE) (mean ± SD) (µm)	142.4 ± 13.3	146.4 ± 13.0	** < 0.01**	4.0, 2.2 to 5.7
Retinal venular calibre (CRVE) (mean ± SD) (µm)	213.1 ± 16.8	217.9 ± 18.3	**< 0.01**	4.8, 2.7 to 7.2

Significant values are in bold.

#### Arteriolar and venular calibres

**Figure 1:**
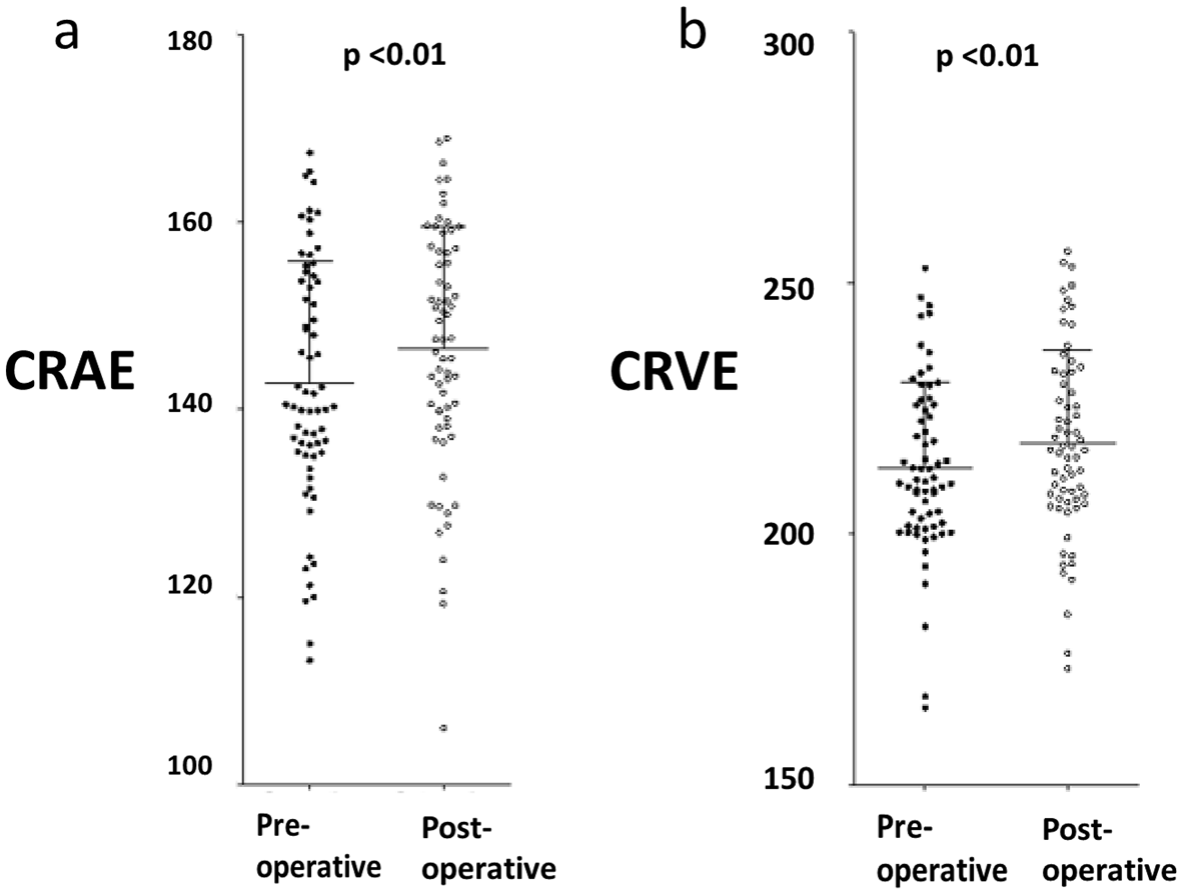
Arteriole (CRAE) and venular (CRVE) calibre pre and post-operatively.

Mean CRAE (arteriolar calibre) increased post-operatively, from 142.4 to 146.4 μm (p < 0.01). The mean CRVE (venular calibre) increased from 213.1 to 217.9 μm (p < 0.01) (Fig. 1).

The effect of surgery on calibre was not uniform (Suppl Fig. 1). CRAE increased in 49 of the 68 patients studied (72%), and decreased by a median of 2.45 um (range 0.5 to 11.8) in the others. The CRVE increased in 48 patients (71%), and decreased by a median of 6.8 um in the others (range 0.27 to 26.24).

#### Major or Other surgery

Individuals undergoing major surgery were more likely to be male (p = 0.01), but were not different in age, nor in their likelihood of having hypertension, diabetes or dyslipidemia than those undergoing Other surgery (Table 3). Individuals undergoing major surgery demonstrated a greater change in CRP (p = 0.01), a higher post-operative CRP (p < 0.01), and a greater change in serum albumin (p = 0.003) and a lower post-operative serum albumin level (p < 0.0001). However their arteriole and venular calibre did not increase more than in individuals undergoing Other surgery (p = 0.64, p = 0.76).

**Table 3:** Participant characteristics with Major surgery (laparotomy) or Other surgery.

	Major surgery (n = 14)	Other surgery (n = 54)	p-value	Difference, 95% CI
Age (mean, SD, years)	56.3 ± 16.1	54.7 ± 14.1	0.71	1.6, − 7.1 to 10.3
Male gender	9 (64%)	14 (26%)	**0.01**	
Hypertension	6 (43%)	20 (37%)	0.76	
Diabetes	0	6 (11%)	0.33	
Dyslipidemia	4 (29%)	12 (22%)	0.73	
Post-operative CRP (mg/L)	76.4 ± 64.8	31.6 ± 46.2 (n = 34)	** < 0.01**	44.8 (11.5 to 78.1)
Change in CRP (mg/L)	65.5 ± 63.7 (n = 13)	24.5 ± 46.7	**0.01**	41.0 (10 to 72.0)
Post-operative albumin (g/L)	28.7 ± 4.5	36.1 ± 4.4 (n = 34)	** < 0.0001**	− 7.4 (− 10.2 to − 4.6)
Change in albumin (g/L)	− 7.6 ± 3.3	− 3.6 ± 4.5	**0.003**	− 4.0 (− 6.6 to − 1.4)
Preoperative CRAE	131.0 ± 10.2	145.4 ± 12.5	**0.0002**	− 14.4 (− 321.6 to − 7.2)
Change in CRAE	5.3 ± 11.7	3.6 ± 12.1	0.64	1.7 (− 5.5 to 8.9)
Preoperative CRVE	206.6 ± 16.8	214.7 ± 16.6	0.11	− 8.1 (− 18.1 to 1.9)
Change in CRVE	3.7 ± 18.8	5.3 ± 17.0	0.76	− 1.6 (− 12.0 to 8.8)

Significant values are in bold.

Explanations for the lack of microvascular dilatation with major surgery include the higher proportion of males (p = 0.01) and the lower pre-operative CRAE (p = 0.002) in this cohort which may have reflected a less flexible microvasculature. The situation was further complicated by the post-operative mean arterial pressure that was higher in participants who underwent major surgery (88.4 ± 10.9 and 83 ± 9.3, p = 0.05).

#### Causes of change in calibre

Arteriolar calibre was wider in participants who were younger (β = − 0.43, 95% CI 0.24 to 0.62, p < 0.01), had a lower mean arterial pressure (β = − 0.61, 95% CI 0.33 to 0.88, p < 0.01), lower CRP levels (β = − 0.07, 0.002 to 0.14, p = 0.04), higher serum albumin levels (β = 1.08, 0.43 to 1.72, p < 0.01) or wider venular calibre (β = 0.49, 0.36 to 0.61, p < 0.01) (Table 4). Hypertensive individuals had a smaller arteriolar calibre (β = − 7.05, 0.78 to 13.32, p = 0.03). Multiple linear regression analysis for post-operative CRAE demonstrated that venular calibre (β = 0.35, 95% CI 0.18 to 0.52 p < 0.01), mean arterial pressure (β = − 0.42, 95% CI 0.10 to 0.73 p = 0.01) and serum albumin level (β = 0.62, 95% CI 0.07 to 18, p = 0.03) were independent predictors of arteriolar calibre.

**Table 4:** Predictors of vessel calibre (CRAE and CRVE) using univariate analysis.

Predictors	β	p-value	95% CI
**Post-operative CRAE**			
Age	− 0.43	** < 0.01**	**0.24 to 0.62**
Hypertension	− 7.05	**0.03**	0.78 to 13.32
Diabetes	1.10	0.70	− 4.47 to 6.66
Dyslipidaemia	− 4.63	0.21	− 11.99 to 2.73
Smoker	0.80	0.80	− 5.52 to 7.12
CRVE	0.49	** < 0.01**	**0.36 to 0.61**
Mean arterial pressure	− 0.61	** < 0.01**	**0.33 to 0.88**
C-reactive protein	− 0.07	**0.04**	**0.002 to 0.14**
eGFR	0.01	0.97	− 0.43 to 0.44
Albumin level	1.08	** < 0.01**	0.43 to 1.72
**Post-operative CRVE**			
Age	− 0.59	** < 0.01**	− **0.86 to 0.31**
Hypertension	− 11.34	**0.01**	**2.59 to 20.09**
Diabetes	− 1.44	0.72	− 9.30 to 6.42
Dyslipidaemia	− 4.95	0.35	− 15.40 to 5.50
Smoker	3.91	0.38	− 4.97 to 12.78
CRAE	0.97	** < 0.01**	0.72 to 1.22
Mean arterial pressure	− 0.70	** < 0.01**	0.29 to 1.11
CRP	− 0.02	0.70	− 0.12 to 0.08
eGFR	0.30	0.34	− 0.33 to 0.93
Albumin level	1.20	**0.02**	0.21 to 2.19

Significant values are in bold.

Venular calibre was wider in participants who were younger (β = − 0.59, − 0.86 to 0.31, p < 0.01), had lower mean arterial pressure (β = − 0.70, 0.29 to 1.11, p < 0.01), higher serum albumin levels (β = 1.20, 0.21 to 2.19, p = 0.02) and wider retinal arterioles (β = 0.97, 0.29 to 1.11, p < 0.01). Venular calibre was smaller in hypertensive individuals (β = − 11.34, 2.59 to 20.09, p = 0.01) (Table 3). Multiple linear regression analysis found that arteriolar calibre was the only independent predictor of venular calibre (β = 1.03, 95% CI 0.71–1.34, p < 0.01).

## Discussion

Surgery is a major stimulus to inflammation which is reflected in the accompanying increased peripheral white blood cell and neutrophil counts, and elevated CRP levels^32^. This study demonstrated that surgery also results in microvascular dilatation of the retinal and, presumably other systemic arterioles and venules. Both arteriole and venular calibre increased after surgery and calibre changes were evident until at least the third post-operative day. The fall in mean arterial pressure at review was also consistent with systemic vasodilatation^32^.

Arteriole and venular calibre usually have multiple determinants and are interrelated. In this cohort undergoing surgery, arteriole widening depended on age, hypertension history, mean arterial pressure, CRP, albumin levels and venular calibre, but multivariate analysis suggested that the only independent determinants of arteriole calibre were mean arteriolar pressure, albumin level and venular calibre. Venular calibre widening depended on age, hypertension history, mean arterial pressure, albumin levels and arteriolar calibre, but again the only independent determinant of venular calibre appeared to be arteriolar calibre.

The study comprised typical hospital patients, including some with hypertension, diabetes, dyslipidemia and a smoking history. The study design of retinal small vessel calibre before and after surgery in individual participants was chosen to examine the effect of surgery per se and to control for long-standing determinants of calibre such as age, diabetes, hypertension and smoking. The study demonstrated an increase in calibre over a short period of time post-operatively. Examining retinal calibre much later after surgery was unlikely to have detected the change due to surgery itself and would have included confounders such as hypertension and diabetes control during this period.

Surgery was associated with an overall increase in CRP, and a fall in serum albumin and Hb that were consistent with inflammation. Interestingly, the microvascular calibre increase did not appear to depend on more extensive surgery despite the higher CRP and lower serum albumin reflecting the greater inflammation associated with laparotomy. Most surgery was minor with only 20% of participants undergoing a major procedure. However the major and Other types of surgery overlapped since it was not possible to directly assess the amount of tissue damage and some so-called major surgery was minor, and some Other surgery, such as breast cancer resection, was more extensive.

There were however other differences in the cohorts with those undergoing major surgery more likely to be male, with smaller calibre arterioles and venules pre-operatively. They also had a higher post-operative mean arterial pressure. These factors may have all contributed to the inability to demonstrate more dilatation with major rather than Other surgery.

Venular dilatation commonly occurs in other forms of inflammation such as diabetes, obesity, dyslipidemia, and rheumatoid arthritis^17–19,33^. Dilatation occurs too with hypoxemia in chronic obstructive pulmonary disease and in smokers^34^.

Previous studies have confirmed that venular dilatation correlates with increased CRP levels^18^. However this study found no direct relationship between the larger retinal small vessel calibre and an increase in CRP or reduction in serum albumin levels. CRP itself might not represent an accurate marker of the inflammation associated with surgery since it depends on the size of the incision of the skin and subcutaneous tissue, IL6 production by local macrophages and CRP production by a healthy liver. Our results suggest that although the small vessel calibre clearly increased after surgery, the causes may have been complex and due to by multiple factors including inflammation and leaky vessels rather than increased CRP.

Indeed we have previously studied retinal small vessel dilatation and CRP levels in a cohort with bacterial infections before and after antibiotic treatment^35^. Again the increased calibre did not correlate directly with the CRP level but rather with WBC counts. Interestingly the mean CRP of treated patients in that study at follow-up was still greater than the CRP seen here after surgery.

There are other explanations for the lack of correlation between increased vessel calibre and CRP levels. These include less compliant less compliant vessels in the participants who were older and had major surgery.

However dilatation is also found in response to fluid loss^20^. After dialysis the microvascular response to fluid shifts of even several litres is transient and retinal small vessel dilatation resolves within hours. The underlying mechanism is probably nitric oxide release by endothelial cells^20^. None of the study participants had a major fall in BP post-operatively and none needed fluid resuscitation after discharge from the recovery ward. It seems unlikely then that fluid loss alone explained the persistent dilatation in relation to major surgery. Other studies have also excluded reduced cardiac output and lower haemoglobin level as causes^36,37^.

The strengths of this study were its novelty, its high recruitment rate and its use of robust and reproducible measures of microvascular calibre. The study was sufficiently large to demonstrate differences in calibre before and after surgery, but a larger study with cytokine measurements might indicate whether the calibre is determined by the levels of inflammatory mediators. The study’s main limitations were the variation in the types of surgery, and the variable and later post-operative time points at which retinal photographs were taken. The increase in calibre may have been more pronounced if the follow-up retinal images were taken earlier, but many participants were too frail immediately post-operatively for retinal photography.

These results are further confirmation that recent surgery affects vessel calibre and that the change in calibre is probably inflammatory in nature. The change in calibre may be a useful model for surgical inflammation, but does not appear to correlate with increased CRP or the fall in serum albumin levels. In other studies, retinal arteriole and venular calibre changes predict cardiac disease, cardiac events and stroke. The use of small vessel calibre to predict risk in hospital patients must take into account confounders such as recent surgery as well as infections.

## Supplementary Information

Supplementary Information.

## Data Availability

All deidentified data used and analysed in the current study is available from the corresponding author on reasonable request.
