# Supplementary figures and images for: Retinal small vessel dilatation in the systemic inflammatory response to surgery
Source: Sci Rep. 2022 Aug 2;12:13291. doi: 10.1038/s41598-022-17467-7 (PMC9346005; doi:10.1038/s41598-022-17467-7)

## Slide 1
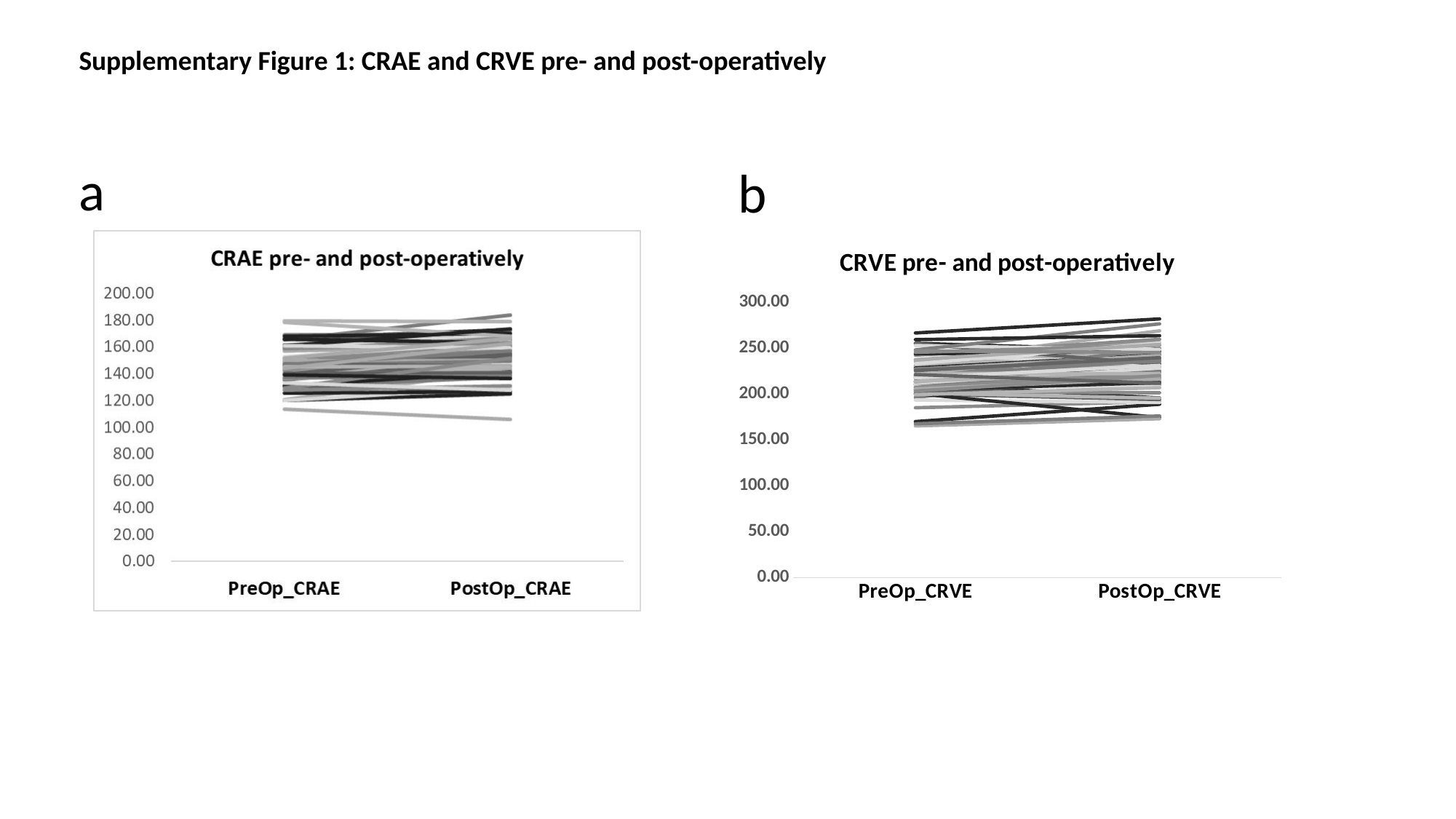

Supplementary Figure 1: CRAE and CRVE pre- and post-operatively
a
b
[unsupported chart]

Supplement: Supplementary file 1 — Supplementary Information. [file 41598_2022_17467_MOESM1_ESM.pptx]
